# Feline Chronic Gingivostomatitis Diagnosis and Treatment through Transcriptomic Insights

**DOI:** 10.3390/pathogens13030192

**Published:** 2024-02-21

**Authors:** Maria Soltero-Rivera, Claire Shaw, Boaz Arzi, Milinda Lommer, Bart C. Weimer

**Affiliations:** 1 Department of Surgical and Radiological Sciences, University of California, Davis, CA 95616, USA; barzi@ucdavis.edu (B.A.); mjlommer@ucdavis.edu (M.L.); 2 Department of Population Health and Reproduction, 100K Pathogen Genome Project, University of California, Davis, CA 95616, USA; clashaw@ucdavis.edu; 3 Aggie Animal Dental Center, Mill Valley, CA 94941, USA

**Keywords:** feline, dentistry, gingivitis, stomatitis, transcriptomics, biomarkers, MSC, stem cells

## Abstract

Feline chronic gingivostomatitis (FCGS) is a debilitating inflammatory oral mucosal disease with a multifactorial etiology. The clinical diagnosis of FCGS is made based on inspection of severe inflammatory lesions and histological confirmation rather than a molecular diagnostic outcome. This gap limits the ability to provide an early diagnosis. In this report, we seek to provide additional diagnostic tools using genomics to aid in providing clinically relevant information. The use of in-depth diagnostic tools, like transcriptomics of diseased tissues, to diagnose FCGS and stratify patients into predictive treatment response groups would dramatically improve both clinical decisions and patient outcomes. In this study, we addressed the gap in diagnostic options using transcriptomic analysis of caudal oral mucosal swab specimens coupled to detailed medical record linkage of FCGS-affected cats undergoing tooth extractions and in some cases administration of mesenchymal stromal cells (MSCs). To better identify markers of disease and potential response to treatment, the transcriptomes of FCGS-afflicted cats were compared to those of healthy cats and those with chronic periodontitis to clearly establish diagnostic biomarker signal transduction connections. Phosphatidylinositol 3-kinase/Ak strain transforming (PI3K/AKT) and stress-activated protein kinases/Jun N-terminal kinase (SAP/JNK) signaling pathways were significantly differentially regulated in FCGS-afflicted cats. Activation of these pathways also differed in the treatment response groups. In conjunction, the enzymes Caspase 4 (CASP4), matrix metalloproteinase-8 (MMP8), and prostaglandin-endoperoxide synthase 2 (PTGS2) were identified as potential biomarkers for the prediction of treatment response outcomes. The observations in the case study support the use of transcriptomics of FCGS patients to contribute to improved molecular diagnostics for the diagnosis and treatment of FCGS.

## 1. Introduction

Feline chronic gingivostomatitis (FCGS) is a devastating, complex, and chronic inflammatory mucosal disease that affects up to 26% of domestic cats. It causes severe oral pain and can be potentially life threatening in at least 10% of cases [1,2]. The current etiologic theories for FCGS include an immune response mediated by CD8+ T cells [3], chronic infection with feline calicivirus (FCV), and induction of IL6, IL17 and pathways related to cells of the myeloid lineage involved in the innate immune response [4,5,6]. Furthermore, co-infection of FCV and puma feline foamy virus (PFFV) as well as infection with feline leukemia virus (FeLV) have been associated with a lack of response to treatment [4,7]. As a naturally occurring disease with an unknown origin, FCGS presents as a particularly intriguing disease to study the role of bacteria and viruses both locally in the oral cavity and more systemically throughout the host.

Though scientifically intriguing, FCGS is a frustrating disease for patients, clients, and clinicians alike. Dental extraction of all or nearly all teeth is the current recommended treatment, and between 1/3 and 1/2 of cats with FCGS will not respond to extraction as the main treatment [5,8]. The treatment protocol for extractions has historically qualified patients as refractory after no clinical response is seen for six months after extraction therapy. For these patients who qualify as refractory, the application of stromal cell therapy has successfully reduced the number of non-responsive cases from ~1/3 and 1/2 to just ~10%. Though the application of mesenchymal stromal cells (MSCs) is effective at reducing disease outcomes, the waiting time to be considered eligible for advanced treatment like MSCs and the lack of other viable treatment options lead to poor long-term prognoses [1,2]. The advent of better diagnostic tools for the identification and treatment of FCGS are therefore necessary for the development of better FCGS care protocols and improved patient outcomes.

Despite the current lack of diagnostic tools to aid in the clinical diagnosis of FCGS in early stages, there are a few tools available to clinicians for the monitoring of disease progression and typical protocols for the application of treatment methods. A standardized activity index, the Stomatitis Disease Activity Index (SDAI), was developed by Dr. Jamie Anderson to monitor clinical manifestations related to oral inflammation in FCGS-afflicted cats. Modified in previous studies [3,9], the SDAI serves as a valuable diagnostic and monitoring tool as it considers both the owner’s assessment of the patient’s quality of life at home and the veterinary practitioner’s gross evaluation of the oral cavity. However, disparities between owner and clinical assessments may arise.

To date, there is no precise way to predict which patient will respond to surgical treatment (which consists of near full-mouth to full-mouth extractions), and nearly 1/3 to 1/2 of patients do not respond, requiring additional medical or regenerative (stromal cell) management. Pre-operative evaluation should include a thorough examination of parameters, such as complete blood count and a serum biochemistry panel. Additionally, viral disease testing for FeLV, feline immunodeficiency virus (FIV), FCV, and PFFV is crucial due to its potential prognostic significance [4,7]. Virus isolation and RT-PCR techniques using samples from the conjunctiva or oropharynx are recommended to enhance FCV detection rates. Despite its potential significance in disease manifestation, there is no currently available commercial test for PFFV. Furthermore, data supporting microbiome shifts in various niches of the oral cavity of FCGS-affected cats support the potential of microbiome markers of disease that could have diagnostic and prognostic significance [10,11]. The availability of a symptom monitoring scheme and some clinically available viral testing provides clinicians with much needed data for the development of a treatment plan, but the lack of more robust methods for the prediction of treatment response and disease development reduces patient outcomes and leaves a noticeable diagnostic gap.

Transcriptomics, the analysis of cellular activity using RNA sequencing, is a tool that may aid in the identification of FCGS disease status and perhaps in prediction of the treatment response. Transcriptomic analysis of FCGS in past studies has focused on affected tissues collected from treatment-naïve patients. Though critical to improving our understanding treatment outcomes, few patients undergoing extractions have been assessed using this sequencing approach to follow disease and treatment progress [3,6]. There is a great need to better understand the cell biology events involved in the pathogenesis of FCGS, especially as they relate to treatment response success. Without the utilization of modern-day sequencing technology, like RNA sequencing, we remain in the dark about specific cellular responses that underlie disease progression and what signaling pathways mediate treatment response. While many approaches exist for the profiling of host cellular activity, RNA sequencing provides an unparalleled look into the activity of the host tissues, and the concurrent use of predictive software allows for the projection of activation and repression at the level of canonical pathways. Such detailed insights may be critical to finally identifying the origins of FCGS, as previous approaches have yet to put forth a consensus on the etiology of this disease. Likewise, the more detailed understanding of host function afforded by this transcriptomic approach supports the development of more personalized treatment plans in the clinical setting, potentiating improved outcomes for afflicted patients and decreased guesswork on the part of clinicians.

In this study, we applied RNA sequencing to a cohort of diseased cats, hypothesizing that cats with FCGS have significantly different transcriptomic signatures that can aid in treatment stratification and optimization of patient outcomes. Using transcriptomic analysis, we tested this hypothesis with samples before and after treatment (i.e., full-mouth extractions or administration of MSCs) of FCGS and used cats with chronic periodontitis as well as healthy cats as controls. Furthermore, the samples collected within a series of patients were obtained from buccal mucosa swabs and were therefore easily obtained samples as compared to histological biopsies or subgingival samples. The ability to use non-invasive sampling methods, as done here, supports the viability of this sequencing technology for diagnostic settings, where attaining biopsies may be costly and requires anesthesia.

Herein, we report functional genes and biological pathways that were found to be differentially expressed when comparing buccal swabs from control cats with FCGS patients. Within the affected patients, we also compared those that responded to full-mouth extractions with those patients that did not respond to extraction treatment and characterized the pathways involved in the response to the administration of adipose-derived mesenchymal stromal cells. The transcriptomic data coupled to patient outcomes strongly support the administration of MSCs in these cases of severe oral inflammation as an effective treatment for a subset of FCGS-afflicted cats and more broadly suggest that such transcriptomic data may be applied in clinics as diagnostics for the identification of disease and prediction of effective treatment.

## 2. Case Description

**Figure 1:**
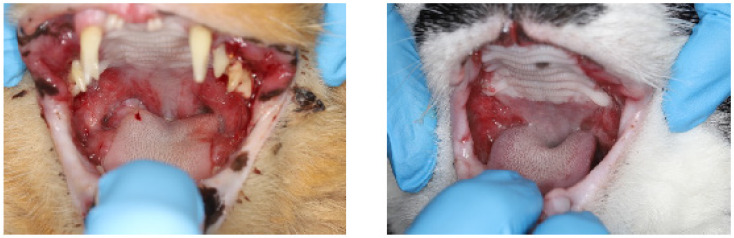
Clinical pictures of feline chronic gingivostomatitis (FCGS)-afflicted oral cavities. (**Left**) Manifestation of FCGS at time of diagnosis with all teeth present. (**Right**) FCGS oral lesions in refractory patient despite undergoing full-tooth extractions.

A total of 42 privately owned cats that were presented for dental care at one of three Northern California Veterinary Centers were included in the study. Consent for study enrollment was obtained from the owner of each cat prior to study initiation. All study procedures were reviewed and approved by the University of California-Davis Institutional Animal Care and Use Committee (IACUC). All owners signed an informed consent prior to sample acquisition. For a detailed overview of patient demographics, disease, and treatment details, please refer to Fried et al. [4]. The study population included 14 clinically normal cats, five cats with mild to moderate periodontitis (also considered as controls), and 23 cats with feline chronic gingivostomatitis (FCGS). A diagnosis of FCGS was made based on moderate to severe inflammation of the oral mucosa lateral to the palatoglossal folds, with or without gingivitis, and was confirmed by histologic evaluation of oral lesions (Figure 1). Cats with severe periodontitis, osteomyelitis of any oral structure, and evidence of oral neoplasia were excluded from this work, as were cats undergoing immunosuppressive treatment and cats with immunocompromising diseases (i.e., diabetes mellitus).

For individuals that were healthy or had periodontitis, an oral mucosal swab specimen was obtained during routine periodontal treatment. For cats with FCGS, swab specimens of the oral mucosal lesions were obtained immediately before tooth extraction (n = 12) or initiation of mesenchymal stromal cell (MSC) therapy after it was determined that the FCGS-affected cat had not responded to tooth extraction (n = 11) [4]. Paired, left and right samples were obtained for one cat in the control group, three in the extraction group and one cat in the mesenchymal stromal cell therapy group.

Briefly, FCGS patients included in the study underwent a pre-operative evaluation including but not limited to a physical and oral examination, complete blood count and serum biochemistry panel, as well as retroviral testing (i.e., feline leukemia virus and feline immunodeficiency virus). Intra-operative evaluation included biopsy of affected buccal mucosa for histopathologic evaluation, lesion characterization, periodontal charting, and full-mouth intraoral dental radiography. Surgical management involved extraction of all teeth (i.e., full-mouth extractions). Patients were then closely monitored for six months to assess the response to extraction therapy. A different population was considered for mesenchymal stromal cell therapy. These were cats that did not show a response to full-mouth extraction treatment six months post-surgery and thus were considered refractory, and these cats were then treated with mesenchymal stromal cell therapy. Mesenchymal stromal cells were adipose-derived allogeneic cells, and each cat received two intravenous injections of 20 million MSCs one month apart [12]. This population was also monitored for six months to assess response to MSC therapy.

**Figure 2:**
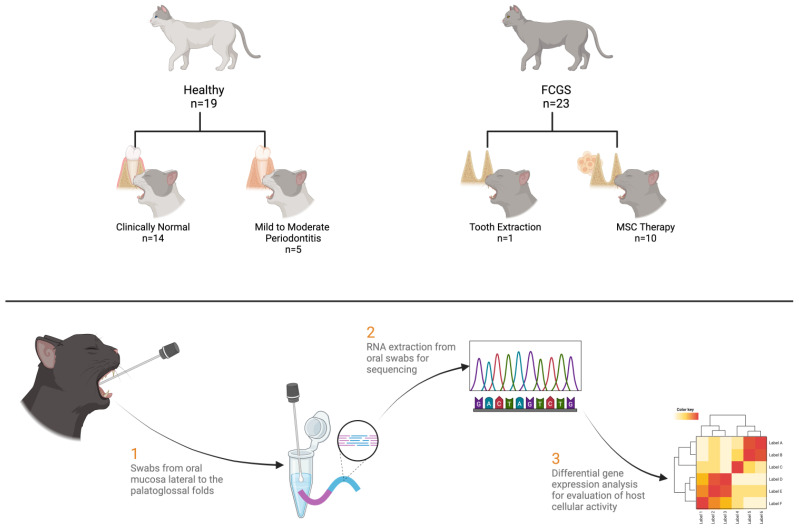
Method schematic detailing the sample numbers and basic collection and sequencing scheme. A total of 42 unique samples were collected, with 9 more samples derived from the same sampling population at contralateral locations or times. Healthy cats were those with no diagnosed dental disease or with mild to moderate periodontitis. Feline chronic gingivostomatitis (FCGS) cats were those with gingivostomatitis and underwent either tooth extraction or mesenchymal stromal cell (MSC) treatment after not responding to extractions. Swabs were taken from the oral mucosa lateral to the palatoglossal folds. mRNA was extracted from the swabs, sequenced, and analyzed.

For each cat, a sterile cotton-tipped applicator was used to swab the oral mucosa lateral to the palatoglossal folds. The swab was placed in a sterile conical tube that contained 250 μL of guanidinium thiocyanate. The tube and its contents were then vortexed before being frozen and stored at –20 °C until transported on dry ice to a laboratory, where the specimen was stored frozen at –80 °C until processed. Samples were processed for deep metatranscriptomic sequencing, aligned to the cat reference genome (felCat9.2_X; GCA_000181335.6), and used to identify specific cat transcripts after removal of the microbiome sequencing reads. The relative abundance of the host differentially expressed genes was determined and compared between FCGS-affected cats and control cats and between FCGS-affected cats that did and did not clinically respond to treatment (i.e., full-mouth extractions or mesenchymal stromal cell therapy). Functional analysis was carried out using Ingenuity Pathway Analysis (IPA), a very highly curated database of signaling pathways and biological information using enrichment analysis approaches as shown in the results section. Gene set enrichment was considered significant when adj *p* < 0.05. Using this approach provided a powerful method to bring biological insight to many intersecting pathways that have multiple interactions and disease associations (Figure 2).

## 3. Results

### 3.1. Feline Calicivirus Status

Feline calicivirus (FCV) has been previously associated with feline chronic gingivostomatitis (FCGS), so FCV prevalence was examined in the population of cats described here. The prevalence of FCV in this feline population was determined using normalized read counts from ultra-deep RNA sequencing (>150 million read/sample) in a previously published work [4]. No cats in the healthy group had detectable levels of FCV, while 21 of 23 cats with FCGS had detectable levels of FCV [4]. Though FCV appears to have a positive association with FCGS disease status in this cohort, it is important to note the detection of FCV varies by method, and a causative role for FCV in FCGS is yet to be determined. One study noted FCV signatures in the genetic material but was unable to find viral particles using immunohistochemistry or in situ hybridization [6], indicating that FCV presence is nuanced and identification of FCV may require multiple methods for verification of infection status.

### 3.2. Regulation of Gene Expression in FCGS

**Figure 3:**
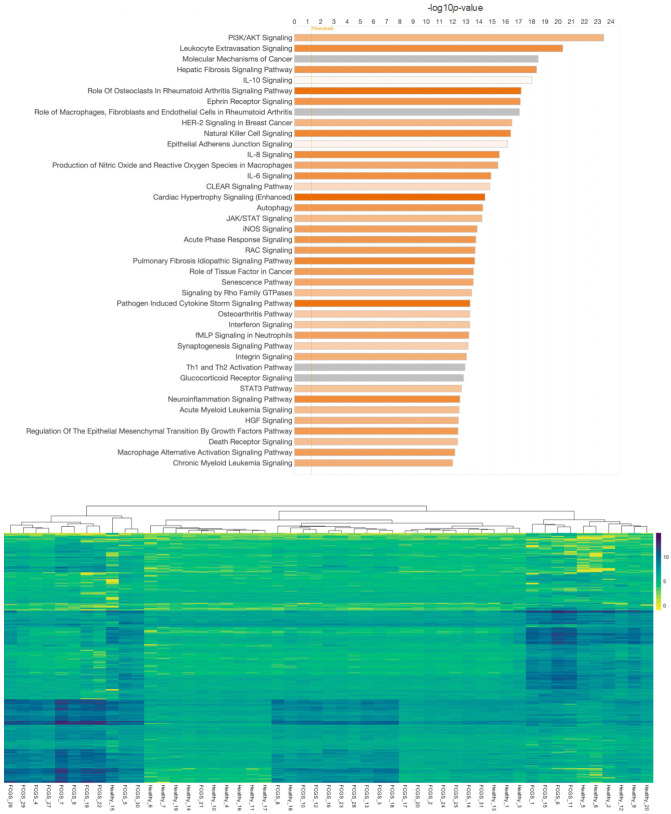
Overview of differentially expressed genes in cats with feline chronic gingivostomatitis (FCGS) compared to healthy controls. IPA (Ingenuity Pathway Analysis) was used to determine the top significantly (−log10*p* > 10) expressed pathways in FCGS patients as compared to healthy samples (**top**). Coloration represents the Z-score of each pathway; orange denoting positive values and gray showing no clear activity pattern in that pathway, with color intensity positively correlated to Z-score. General expression patterns across healthy and FCGS samples were also evaluated and displayed using a heatmap (**bottom**). R (version 4.2.2) package pheatmap (version 1.0.12), with Euclidean distance clustering of both genes and samples with log2 normalized read counts as input data was used to generate the map.

The potential association of FCV but lack of a mechanistic confirmation led us to consider that host regulation of cellular pathways may instead be an effective diagnostic tool for the diagnosis of FCGS [6] and subsequent stratification into predictive response groups. To explore these potentially relevant changes in host biological function, the metatranscriptomes of FCGS cases were compared to healthy controls. Gene expression analysis of gene induction and repression patterns between healthy and FCGS patients revealed notable individual variation in expression patterns, but, interestingly, this analysis displayed some commonality in repression patterns between FCGS-afflicted and healthy cats. Using the significantly different (adj. *p*-value < 0.01) expression changes of entire biological pathways, we found notable differences between the diseased and healthy groupings. The most significantly modified pathways (adj. *p*-value < 0.01) expressed in FCGS patients as compared to healthy samples were found in multiple signal transduction pathways that are known to be associated with inflammatory diseases (Figure 3). Phosphatidylinositol 3-kinase/Ak strain transforming (PI3K/AKT) signaling was significantly induced in FCGS patients compared to healthy controls (bias-corrected Z-score = 3.812, adj. *p*-value = 3.84 × 10^−24^). Leukocyte extravasation (adj. *p*-value = 4.49 × 10^−21^), hepatic fibrosis (adj. *p*-value = 4.30 × 10^−19^), role of osteoclasts in rheumatoid arthritis (adj. *p*-value = 6.40 × 10^−18^), ephrin receptor (adj. *p*-value = 7.94 × 10^−18^), natural killer cell (adj. *p*-value = 3.95 × 10^−17^), IL8 (adj. *p*-value = 2.86 × 10^−16^), IL6 (adj. *p*-value = 1.24 × 10^−15^), cardiac hypertrophy (adj. *p*-value = 3.51 × 10^−15^), and autophagy signaling pathways (adj. *p*-value = 5.15 × 10^−15^) also showed strong activation (bias-corrected Z-score > 3.50).

### 3.3. Treatment Effect on Gene Expression Patterns

**Figure 4:**
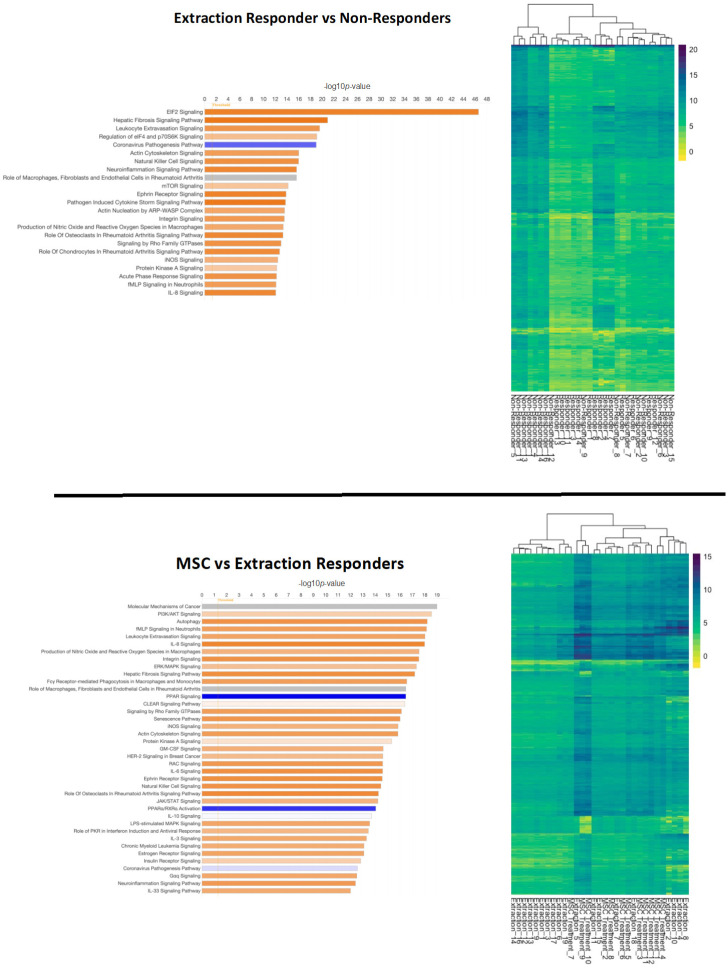
Overview of differentially expressed genes in patients that responded to extractions (**top**) and those that underwent mesenchymal stromal cell (MSC) therapy (**bottom**). Ingenuity Pathway Analysis (IPA) was used to determine the top significantly (−log10*p* > 10) expressed pathways in extraction responsive vs. non-responsive cats and MSC-treated vs. extraction-responding cats. Coloration represents the Z-score of each pathway; orange denoting positive values, blue denoting negative values, and gray showing no clear activity pattern in that pathway, with color intensity positively correlated to Z-score. General expression patterns across healthy and feline chronic gingivostomatitis (FCGS) samples was also evaluated by heatmap. R (version 4.2.2) package pheatmap (version 1.0.12) with Euclidean distance clustering of both genes and samples with log2 normalized read counts as input data was used to generate the map.

Noting the differences in the broad comparison of healthy and diseased cats, we sought to further differentiate patients by their response to tooth extractions and in some cases follow-up mesenchymal stromal cell (MSC) therapy if extraction therapy was not successful in resolving the disease. Through a detailed comparison of these groups, we were able to identify key gene expression regulation that distinguishes the treatment response groups from one another at a cellular expression level. Pathways related to cellular signaling, immunity, inflammation, and disease processes were dysregulated in the other two comparisons performed. The top significantly (adj. *p*-value < 0.01) expressed pathways in patients that responded to extractions and those that underwent MSC therapy (i.e., non-responsive to extractions) were further evaluated (Figure 4).

Eukaryotic Initiation Factor 2 (EIF2) signaling was markedly activated in patients that responded to extractions (bias-corrected Z-score = 6.062, adj. *p*-value = 2.62 × 10^−42^). Hepatic fibrosis (adj. *p*-value = 1.21 × 10^−21^), leukocyte extravasation (adj. *p*-value = 2.73 × 10^−20^), natural killer cell (adj. *p*-value = 1.05 × 10^−16^), neuroinflammation (adj. *p*-value = 2.3 × 10^−16^), ephrin receptor (adj. *p*-value = 1.41 × 10^−14^) and pathogen-induced cytokine storm signaling pathways (adj. *p*-value = 1.75 × 10^−14^) were also strongly activated (bias-correct Z-score > 3.0) in cats that responded well to extractions. The coronavirus pathogenesis pathway was inactivated in extraction-responsive patients (bias-corrected Z-score = −2.41, adj. *p*-value = 1.04 × 10^−19^). MSC responders compared to extraction responders activated pathways for autophagy (adj. *p*-value = 6.16 × 10^−19^), IL8 (adj. *p*-value = 1.01 × 10^−18^), integrin (adj. *p*-value = 2.94 × 10^−18^), and hepatic fibrosis signaling pathways (adj. *p*-value = 6.32 × 10^−18^). Notably MSC responders showed significant inactivation of peroxisome proliferator-activated receptor (PPAR) (adj. *p*-value = 3.33 × 10^−17^) and PPARα/ Retinoid X receptor alpha (RXRα) (adj. *p*-value = 8.93 × 10^−15^) activation signaling pathways.

**Figure 5:**
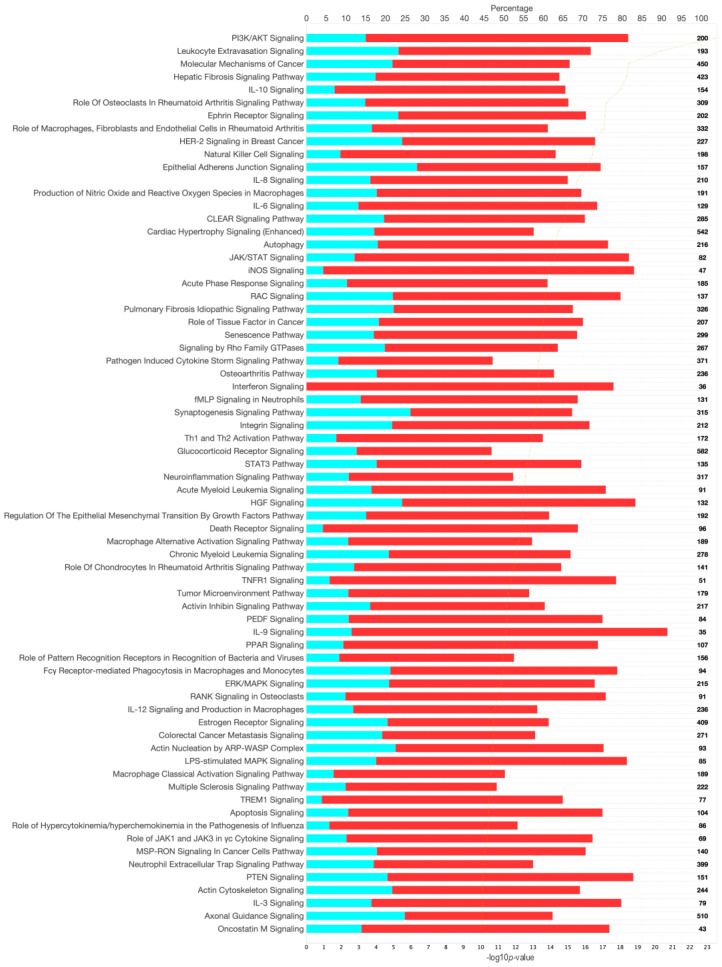
Canonical signaling pathways from gene and pathway enrichment (IPA) comparing feline chronic gingivostomatitis (FCGS) samples to healthy samples. Significance cutoff of -log10*p* >10. The top axis represents the percentage of the pathway annotated in the gene expression data set that was downregulated (teal), upregulated (red), or not represented in the data (white). The total gene count in each pathway is noted on the right-hand side of the figure. The bottom axis represents the significance of each pathway and corresponds to the orange line on each figure, and pathways are ordered from most significant to least from top to bottom.

The induction and repression of canonical signaling pathways was evaluated for each diseased and treatment group comparison: FCGS stomatitis vs. healthy, extraction responsive vs. non-responsive, and MSC responsive vs. non-responsive (Figure 5).

**Figure 6:**
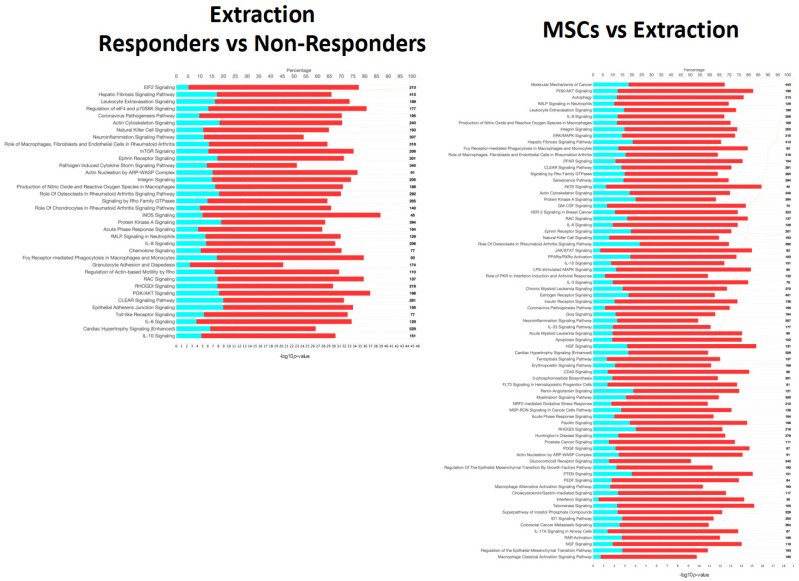
Canonical signaling pathways from Ingenuity Pathway Analysis (IPA) comparing extraction-responsive samples to non-responder samples (**left**) and those that received mesenchymal stromal cells (MSC) treatment after extraction compared to those that received extractions only (**right**). Significance cutoff of -log10*p* >10. The top axis represents percentage of the pathway annotated in the gene expression data set that was downregulated (teal), upregulated (red), or not represented in the data (white). The total gene count in each pathway is noted on the right-hand side of the figure. The bottom axis represents the significance of each pathway and corresponds to the orange line on each figure, and pathways are order from most significant to least from top to bottom.

**Figure 7:**
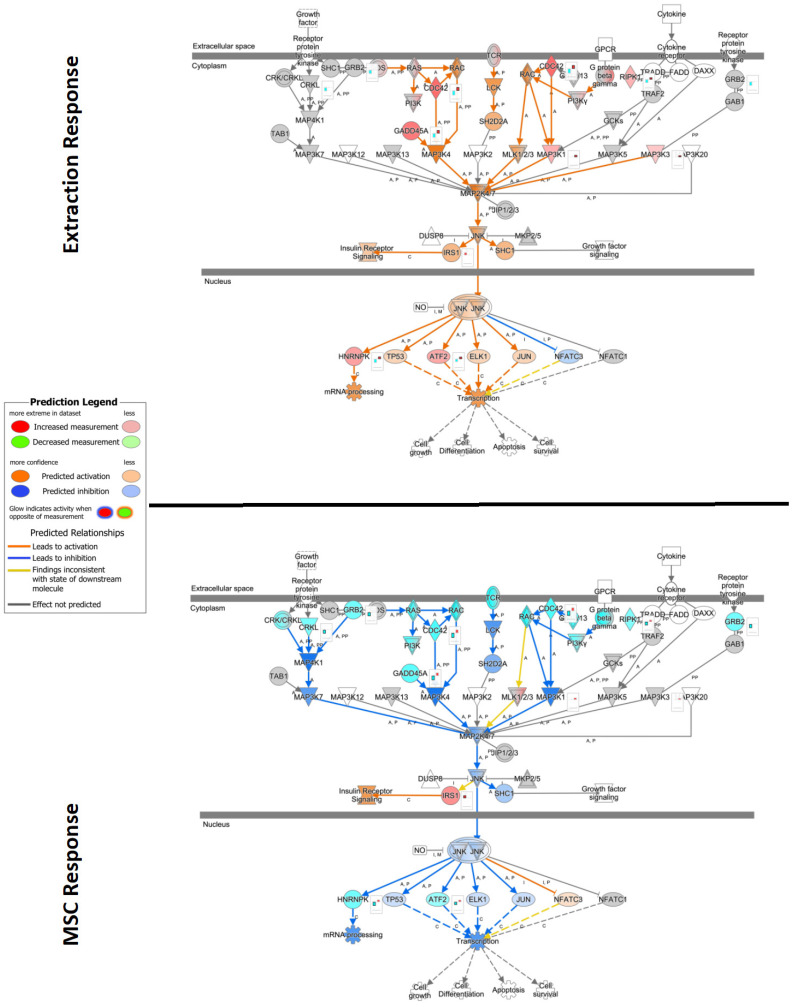
Ingenuity Pathway Analysis (IPA) pathway of stress-activated protein kinases/Jun N-terminal kinase (SAP/JNK) signaling overlayed with gene expression data from extraction and mesenchymal stromal cell (MSC) treatment samples. Significance cutoff of −log10P > 1.3, and data shown as log2 fold change expression. Red represents increased measurement of that gene, while teal indicates decreased measurement. Orange and blue overlays represent predicted activation or inhibition, respectively. (**Top**) Comparison of differentially expressed genes from patients that responded to extraction treatment versus those who had no successful therapeutic response to extractions. (**Bottom**) Comparison of differentially expressed genes from non-responder extractions patients that underwent MSC therapy versus patients that underwent only extraction therapy.

Significant induction (positive bias-corrected Z-score, adj. *p*-value < 0.01) of disease-associated pathways was observed for each comparison. When evaluating individual pathways, extraction- and MSC-responsive patients showed opposite effects on PI3K/AKT signaling, with overall activation noted in extraction-responsive patients and inactivation noted in MSC-responsive patients (Figure 6). A similar effect was observed with stress-activated protein kinases/Jun N-terminal kinase (SAPK/JNK) signaling, with activation noted in extraction-responsive (bias-corrected Z-score = 3.36) and inactivation noted in MSC-responsive patients (bias-corrected Z-score = −3.67) (Figure 7).

### 3.4. Biomarker Analysis

Identification of diagnostic markers, including specific genes that may signal disease susceptibility or predictive treatment response, are lacking in this disease cluster. To address that gap, we examined potential diagnostic biomarkers for each comparison group using the biomarker identification feature in Ingenuity Pathway Analysis (Qiagen, Hilden, Germany). The analysis, which identifies biomarker candidates based on observed pathway regulation and through comparison to a curated database of known biomarkers for other diseases, revealed several candidates associated with a positive response to extraction treatment. Among these, the enzymes Caspase 4 (CASP4), matrix metalloproteinase-8 (MMP8), and prostaglandin-endoperoxide synthase 2 (PTGS2) emerged as putative biomarkers associated with therapeutic outcomes (Table 1). All three biomarker candidates were induced (expression log ratio > 1) in the population of cats that had a positive response to extractions, but MMP8 was the most significantly induced of the three (adj. *p*-value = 0.0032). Notably, CASP4 was also identified as a potential biomarker for cats who responded well to MSC therapy, but the repression rather than induction of CASP4 was important for this cohort. In a similar vein, MMP8 and PTGS2 were also noted as biomarkers for MSC response but like CASP4 are repressed in the MSC responsive group. The identification of these three genes as important markers of patient response to treatment, alongside the clearly divergent expression patterns in extraction responders versus MSC responders, provides an exciting roadmap towards clinical application of such genetic tools.

Developing early diagnostic molecular biomarkers would be very helpful in the clinic as a method to quickly associate therapies and possible trajectory of disease progression. Validation of these markers is valuable and may provide a roadmap to understand the patient’s potential response to treatments and need for adjunctive therapy (i.e., full-mouth extraction with MSC therapy), effectively increasing the success rates and reducing suffering in these patients. Identification of the expression patterns of these genes and their related pathways in larger feline cohorts will be imperative for the future application of these biomarkers as clinical standards for treatment outcome predictions.

## 4. Discussion

Feline chronic gingivostomatitis (FCGS) significantly decreases the quality of life of affected patients [5,8,13]. Despite advances in understanding the cellular processes and signaling pathways associated with FCGS through targeted and untargeted surveys, questions regarding treatment response and outcomes remain unresolved [3,6,14,15,16,17]. This study used shot gun ultra-deep metatranscriptomic analysis to evaluate the host response in FCGS before and after treatment (i.e., full-mouth extractions and mesenchymal stromal cell therapy) using a non-invasive sampling technique, namely, swabs of affected oral mucosa. Our findings revealed distinct patterns in signal pathway activation of FCGS-affected patients versus control patients (i.e., healthy and chronic periodontitis). Given the limited responses of the oral mucosa, these findings underscore the potential of molecular analysis for diagnosing FCGS in veterinary practice. Furthermore, stratification of treatment response groups by the patterns of differential gene expression supports the conceivable use of this technology as a predictive diagnostic tool for more personalized and effective treatment plans in the clinic.

In the work undertaken here, the Phosphatidylinositol 3-kinase/Ak strain transforming (PI3K/AKT) signaling pathway emerged as a significant molecular basis for disease with moderate activation (Z-score = 3.81) in the FCGS-affected group. Constitutive activation of the PI3K/AKT pathway has been observed in inflammatory and autoimmune diseases, viral infections, and autoinflammatory syndromes [18,19,20,21]. PI3K/AKT is known to influence immune cell function, such as the activity of myeloid cell populations, and contributes to the regulation of cytokine production, bringing to light once again the role of the innate immune system in this disease [22]. Additionally, the pathway may play a role in the host cell response to viral infections, influencing viral replication and the antiviral immune response, which also supports the current theory of viral involvement in this disease [4,7,20].

Leukocyte extravasation, hepatic fibrosis, and the role of osteoclasts in rheumatoid arthritis exhibited strong activation, signifying their contribution to the inflammatory and tissue remodeling aspects of FCGS. Hepatic fibrosis implicates the liver’s role in immune regulation [23]. The hepatic fibrosis signaling pathway may also represent a side effect of the medical management utilized in this patients prior to surgical or regenerative interventions [1]. Parallels between rheumatoid arthritis and periodontitis have been historically drawn, and periodontitis is a common feature of FCGS [24]. Ephrin receptor, leukocyte extravasation, and natural killer cell signaling are consistent with previous studies and highlight the involvement of the immune responses and potentially the host defense mechanisms against the disease [3]. Strong activation of IL8 and IL6 cytokine signaling pathways supports the presence of a pro-inflammatory microenvironment, a hallmark of FCGS pathology, and is consistent with previous transcriptomic data [3,6,15,17]. Activation of the cardiac hypertrophy and autophagy signaling pathways may indicate broader systemic effects associated with FCGS, potentially affecting cardiac function and autophagy regulation. Periodontal pathogens have been linked to myocardial hypertrophy in humans but research in this area of feline medicine is lacking [25]. The feline population sampled in this study was not additionally screened for cardiovascular diseases, so the incidence of undiagnosed concurrent heart diseases may likewise contribute to these findings. Systemic effects of FCGS have been previously reported; however, mechanisms to explain how these may specifically affect the cardiovascular system have not been evaluated [3,17,26,27].

When comparing signaling pathways with treatment outcome, a distinct difference in the mechanism by which extractions and mesenchymal stromal cell (MSC) therapy led to a response became apparent. Extraction treatment may lead to a change in the microbiome of affected patients by eliminating the subgingival compartment as a potential source of chronic antigenic stimulation [10,11,28]. Of great relevance, eukaryotic initiation factor 2 (EIF2) signaling showed a marked induction in patients that responded to extraction. Induction of the EIF2 signaling pathway is typically associated with a positive outcome in response to cellular stress, particularly during the integrated stress response. EIF2 plays a crucial role in regulating protein synthesis and cellular adaptation to various forms of stress, including viral infection, endoplasmic reticulum stress, proteostasis and protein misfolding diseases, ischemia/reperfusion injury, cancer, and immunosuppressive therapy [29]. Interestingly, the coronavirus pathogenesis pathway was inactivated in extraction-responsive patients, suggesting an association between FCGS treatment response and the elimination of viral infections that is consistent with previous studies [4,30,31].

MSC therapy led to complete remission in up to 70% of cats with FCGS that had failed full-mouth extractions [12,26,32]. This may be due to their capability in suppressing viral replication and augmenting clearance through enhancement of antiviral immunity [33,34]. Autophagy, IL8, and hepatic fibrosis signaling displayed strong activation in MSC responders, suggesting their potential as therapeutic targets for these cells. Whether MSCs cause activation or inactivation of these peroxisome proliferator-activated receptor (PPAR) and PPARα/Retinoid X receptor alpha (RXRα) activation signaling pathways can depend on the context, the specific type of MSCs, and the target tissue or disease being studied [35]. In our case, PPAR and PPARα/RXRα activation signaling pathways were inactivated in responders to treatment with adipose-derived MSCs. Opposing effects were observed in PI3K/AKT signaling and stress-activated protein kinases/Jun amino-terminal kinases (SAPK/JNK) signaling between extraction and MSC-responsive patients.

Treatment stratification using genetic markers has been proposed for neoplastic, psychiatric, fulminant acute, and chronic inflammatory diseases in humans, among others [36,37,38,39]. In this study, caspase-4 (CASP-4), matrix metallopeptidase 8 (MMP-8), and prostaglandin-endoperoxide synthase 2 (PTGS2) emerged as putative biomarkers indicative of potential therapeutic outcomes. CASP4, also known as inflammatory caspase, plays a role in the regulation of inflammatory responses and cell death, which may be relevant in the context of eliminating infected cells [40,41]. In oral infections, either viral or bacterial in origin, CASP4 may be activated as part of the host’s immune response to clear the infection. However, the specific role of CASP4 in oral and dental diseases is not as extensively studied as some other caspases.

While the relationship between CASP4 and periodontal disease is not yet fully elucidated, another biomarker identified in this study has already been recognized for its role in marking periodontal disease. MMP8 is currently considered to be one of the most promising biomarkers for periodontitis in oral fluids and has demonstrated a high diagnostic precision in distinguishing mild from severe periodontitis sites [42,43]. Our current findings are in support of previous reports of a high prevalence of moderate to severe periodontitis in patients with FCGS [44]. Increased expression of MMP8 has also been shown in patients with oral lichen planus (OLP), a chronic inflammatory oromucosal disease, as compared to healthy patients [45]. OLP and FCGS, though not analogous, share similarities in the inflammatory nature, lymphoplasmacytic inflammation, cytokine involvement, possible genetic basis, and the role of the innate immune system [46]. Considering the cohort of cats used in this biomarker analysis and the treatment schemes, MMP8 was likely the most significantly correlated marker for response to extraction given its known role in periodontitis and the inclusion of cats who had not yet undergone extractions. With the presentation of this disease, cats retaining teeth very likely have moderate to severe periodontitis in conjunction with FCGS lesions and thus exhibited highly correlated MMP8 levels that deviated from treatment cats. All considered, MMP8 stands to be a notable marker for this disease given its established role in other oral diseases.

A third identified biomarker, PTGS2, can be induced in response to inflammation, infection, or tissue injury in the oral mucosa. Elevated PTGS2 expression leads to the pro-duction of prostaglandins, which can contribute to inflammation and pain [47]. While PTGS2 is not a direct driver of tooth resorption, it may indirectly contribute to this process in cases where inflammation is a factor, such as in the approximately 50% of FCGS cats that also have tooth resorption [44]. PTGS2 is expressed in inflamed periodontal tissues, and its activity can contribute to the local inflammatory response in periodontitis. Prostaglandins produced by PTGS2 can enhance inflammation, bone resorption, and tissue destruction in the periodontal area [48]. Additionally, an imbalance between prostaglandins and N-acylethanolamines has been reported in humans with OLP [49].

In summary, this comprehensive analysis provides unique and valuable insights into the molecular mechanisms underlying FCGS and the responses to different treatment modalities. This study focused on large differential differences between treatment groups, and more subtle changes may also be considered biologically relevant depending on the specific function and pathway in which the gene is involved. Considering differential expression and genetic variation in each patient, the potential number of genetic variants that underly FCGS could be larger than observed in this study. This may lead to differences in candidate genes or alleles that additional studies may find due to differences in population sub-structures. Nonetheless, there is strong evidence to support this disease having an infectious component in at least modulating disease progression [4,7,10,11,28] that can be explored in future studies that include the microbiome component of the tissue in cases and controls. The findings reported here contribute to our understanding of this complex disease and may pave the way for more targeted and effective therapeutic approaches in the future. Further research and clinical validation are needed to fully harness the potential of these insights for the benefit of FCGS patients.

## 5. Conclusions

The clinical application of biomarkers to provide an insight into feline chronic gingivostomatitis (FCGS) and its therapeutic outcome will be a valuable tool. The use of transcriptomic technology, as demonstrated here, revealed cellular signaling pathways that were differentially regulated in FCGS-afflicted cats, and activation of these pathways differed in the treatment response groups as well. We identified caspase-4 (CASP4), matrix metalloproteinase (MMP8), and prostaglandin-endoperoxide synthase 2 (PTGS2) as potential biomarkers for the prediction of treatment response outcomes. Together, the differential regulation of cellular signaling pathways and identification of biomarker candidates suggests that transcriptomic analysis of FCGS patients can contribute to improved molecular diagnostics for the identification and treatment of this severe oral inflammatory disease.

## Figures and Tables

**Table 1:** Significantly differential biomarkers associated with the response to extraction defined using IPA biomarker analysis.

Gene Symbol	Entrez Gene Name	Cellular Location	Enzyme Family	Fold Change	*p*-Value	Adjusted *p*-Value (q-Value)
CASP4	Caspase 4	Cytoplasm	Cysteine proteases	1.37	0.000264	0.0172
MMP8	Matrix metallopeptidase 8	Extracellular Space	Zinc- and calcium-dependent endo-peptidases	3.39	0.0000125	0.00324
PTGS2	Prostaglandin-endoperoxide synthase 2	Cytoplasm	Prostaglandin G/H synthase	2.43	0.000101	0.0104

## Data Availability

Please contact the corresponding authors for data presented in this study.
